# Supplementary material for: Generalized Michaelis–Menten rate law with time-varying molecular concentrations
Source: PLoS Comput Biol. 2023 Dec 11;19(12):e1011711. doi: 10.1371/journal.pcbi.1011711 (PMC10735182; doi:10.1371/journal.pcbi.1011711)
Supplement: Attachment — Submitted filename: Response_to_Reviewers_Kim.pdf [file pcbi.1011711.s003.pdf]

Response to Reviewer #1

First of all, we are very thankful for Reviewer's careful comments and suggestions. The Reviewer kindly noted that our work *"presents a novel approach ⋯ Overall, the manuscript offers valuable insights into biochemical model reduction beyond the widely-used QSSA."*

We believe that Reviewer's comments have considerably improved our manuscript. Here, we offer a list of the changes made in the manuscript in accordance with Reviewer's comments.

> *"Major: 1. Page 7, Line 184. The ETS returns to the QSSA in its form when the effective time delay is ignorable. Is the condition for ignoring the effective time delay consistent with the tQSSA validity condition?."*

We appreciate Reviewer's insightful question. The answer to this question is yes, but we want to clarify *how small* the effective time delay should be for the validity of the tQSSA. Text S5 shows that the conditions in Eqs. (S29), (S31), and (S34) should be satisfied for the validity of the tQSSA through more rigorous derivation than the previously-reported condition in Ref. [S3]. Because Eqs. (S29) and (S31) are easy to satisfy (as described in Text S5), we here focus on Eq. (S34) below.

$$\varepsilon_{\mathrm{tQ}}(\tau) \equiv \frac{1}{\bar{C}_{\mathrm{tQ}}(\tau)}\left|\Delta_{\mathrm{tQ}}^{-1}(\tau)\frac{\mathrm{d}\bar{C}_{\mathrm{tQ}}(\tau)}{\mathrm{d}\tau} - \Delta_{\mathrm{tQ}}^{-2}(\tau)\frac{\mathrm{d}^2\bar{C}_{\mathrm{tQ}}(\tau)}{\mathrm{d}\tau^2}\right| \ll 1. \quad \text{(S34)}$$

To gain further insights into Eq. (S34), we assume that molecular levels oscillate with period T and thus

$$\bar{C}_{\mathrm{tQ}}(\tau) \approx \overline{C}_{\max}\left\{1 - \frac{\alpha_{\mathrm{C}}}{2}\left[1 + \cos\left(\frac{2\pi}{k_\delta T}\tau\right)\right]\right\},$$

where $\overline{C}_{\max}$ and α_{C} ($0 < \alpha_{\mathrm{C}} < 1$) denote the peak level of $\bar{C}_{\mathrm{tQ}}(\tau)$ and the peak-to-trough difference of $\bar{C}_{\mathrm{tQ}}(\tau)$ divided by the peak level, respectively, and k_δ is a rate parameter in Eq. (1) in the main text with relation $\tau = k_\delta t$. Using this approximate form of $\bar{C}_{\mathrm{tQ}}(\tau)$, Eq. (S34) is rewritten as

$$\varepsilon_{\mathrm{tQ}}(\tau) \approx \frac{\alpha_{\mathrm{C}}}{2}\frac{1}{1 - \frac{\alpha_{\mathrm{C}}}{2}\left[1 + \cos\left(\frac{2\pi}{k_\delta T}\tau\right)\right]}\left[\frac{2\pi}{k_\delta T\Delta_{\mathrm{tQ}}(t)}\right]\left\{1 + \left[\frac{2\pi}{k_\delta T\Delta_{\mathrm{tQ}}(t)}\right]^2\right\}^{\frac{1}{2}}$$
$$\times\left|\sin\left\{\frac{2\pi}{k_\delta T}\tau - \tan^{-1}\left[\frac{2\pi}{k_\delta T\Delta_{\mathrm{tQ}}(t)}\right]\right\}\right| \ll 1.$$

For $\alpha_{\mathrm{C}} \sim O(0.1)$, the above condition is roughly satisfied when $\left[k_{\delta}\Delta_{\mathrm{tQ}}(t)\right]^{-1} \ll T$. In other words, if the effective time delay {i.e., $\left[k_{\delta}\Delta_{\mathrm{tQ}}(t)\right]^{-1}$} is smaller enough than the time-scale of molecular concentration changes (i.e., T), the tQSSA would likely work. Therefore, we have revised Text S5 by adding the following sentence to the description of Eq. (S34):

> "If molecular concentrations vary over time with a characteristic time-scale of T, Eq. (S34) roughly requires the effective time delay $\left[k_{\delta}\Delta_{\mathrm{tQ}}(t)\right]^{-1}$ in Eq. (6) to be smaller enough than T for the validity of the tQSSA."

Next, the Reviewer made the following suggestion:

> *"2. Text S5. The applicability of the ETS and QSSA were compared with the area of the parameter regions where the validity conditions for the ETS (Eq. (S32)) and QSSA (Eq. (S34)) were satisfied, respectively. Does the validity region for the ETS cover the validity region for the QSSA? Please illustrate the validity regions to provide more information."*

We appreciate Reviewer's valuable question and suggestion. The validity region of the ETS essentially covers that of the tQSSA. Specifically, among physiologically-relevant conditions in Table S3, the conditions with $\max_{\tau}\left[\varepsilon_{\gamma}(\tau)\right] \leq 0.1$ [Eq. (S32)] cover 99.8% of the conditions with $\max_{\tau}\left[\varepsilon_{\mathrm{tQ}}(\tau)\right] \leq 0.1$ [Eq. (S34)]. In the case of TF–DNA interactions, the conditions with $\max_{\tau}\left[\varepsilon_{\mathrm{TF}\gamma}(\tau)\right] \leq 0.1$ [Eq. (S36)] cover all the conditions with $\max_{\tau}\left[\varepsilon_{\mathrm{TFQ}}(\tau)\right] \leq 0.1$ [Eq. (S38)]. Therefore, the validity region of the ETS covers that of the (t)QSSA, in practice.

To further illustrate this point, we have obtained the validity regions by spanning wide ranges of K and k_{δ}, while the other parameter values are fixed. Again, the validity region of the ETS covers that of the (t)QSSA, as presented in new Fig. S1 below. The ETS is particularly more valid than the (t)QSSA for larger K and smaller k_{δ}, which tend to lengthen the effective time delay $\left[k_{\delta}\Delta_{\mathrm{tQ}}(t)\right]^{-1}$.

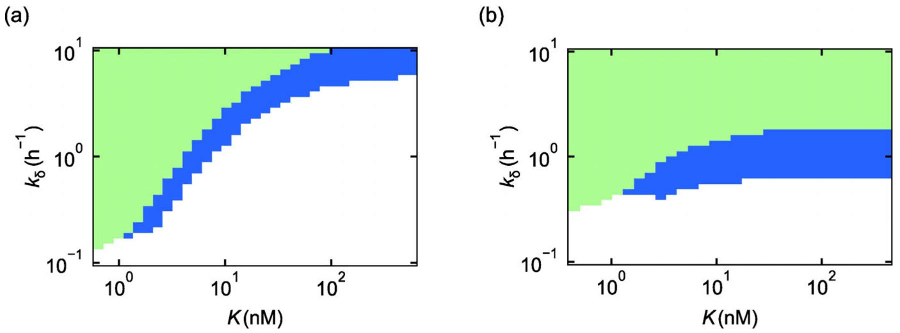

New Fig. S1. Preconditions of rate laws. (a) The ranges of K and k_δ valid for the ETS with $\max_\tau[\varepsilon_1(\tau)] \le 0.1$, $\max_\tau[\varepsilon_2(\tau)] \le 0.1$, and $\max_\tau[\varepsilon_\gamma(\tau)] \le 0.1$ cover the ranges for the tQSSA with $\max_\tau[\varepsilon_{\mathrm{tQ}}(\tau)] \le 0.1$ instead of $\max_\tau[\varepsilon_\gamma(\tau)] \le 0.1$. Green represents the ranges common for both the ETS and tQSSA, and blue represents those only for the ETS. The calculations are based on Eqs. (S4), (S5), (S29), (S31), (S32), (S34), and (S40) (Texts S5 and S7). (b) In the case of TF–DNA interactions, the ranges of K and k_δ valid for the ETS with $\max_\tau[\varepsilon_{\mathrm{TF}}(\tau)] \le 0.1$ and $\max_\tau[\varepsilon_{\mathrm{TF}\gamma}(\tau)] \le 0.1$ cover the ranges for the QSSA with $\max_\tau[\varepsilon_{\mathrm{TFQ}}(\tau)] \le 0.1$ instead of $\max_\tau[\varepsilon_{\mathrm{TF}\gamma}(\tau)] \le 0.1$. Green represents the ranges common for both the ETS and QSSA, and blue represents those only for the ETS. The calculations are based on Eqs. (S21), (S35), (S36), (S38), and (S43) (Texts S5 and S8). In (a) and (b), parameters are selected from Table S3, and their specific values are presented in Table S10.

Therefore, we have added the above Fig. S1, Table S10 for its parameter values, and the following sentences (Text S5) to the revised manuscript:

> "Among them, the conditions with $\max_\tau[\varepsilon_\gamma(\tau)] \le 0.1$ cover 99.8% of the conditions with $\max_\tau[\varepsilon_{\mathrm{tQ}}(\tau)] \le 0.1$, supporting more general applicability of the ETS than the tQSSA's. In addition, Fig. S1(a) shows that the ETS is particularly more valid than the tQSSA for larger K and smaller k_δ, which tend to lengthen the effective time delay $[k_\delta \Delta_{\mathrm{tQ}}(t)]^{-1}$. ⋯ [In the case of TF–DNA interactions] the conditions with $\max_\tau[\varepsilon_{\mathrm{TF}\gamma}(\tau)] \le 0.1$ cover all of the conditions with $\max_\tau[\varepsilon_{\mathrm{TFQ}}(\tau)] \le 0.1$, supporting more general applicability of the ETS than the QSSA's. In addition, Fig. S1(b) shows that the ETS is particularly more valid than the QSSA for larger K and smaller k_δ, which tend to lengthen the effective time delay $k_\delta^{-1}[1 + K^{-1} A_{\mathrm{TF}}(t)]^{-1}$ in Eq. (8)."

The Reviewer made the following comment:

> *"Minor: 1. Figure 1. The model equations and parameters used to draw the graphs were not clearly explained."*

The model equations in Figs. 1(a) and 1(b) are described in Texts S7 and S8, respectively, together with their parameters in Table S7. Therefore, in the revised legend of Fig. 1, we have referred to these sources.

> *"2. Page 5, Line 198–209. The physical interpretation of the ETS is hard to understand. This part needs to be revised to improve readability."*

Following Reviewer's valuable suggestion, we have revised this paragraph in the manuscript, as follows (the revised part in bold):

> "In other words, the less the free molecules, the more the time delay, which is at most k_δ^{-1}. One can understand this observation as follows: $-\boldsymbol{k_\delta C(t)}$ **in Eq. (1) gives the expectation that the decay time-scale (**$\boldsymbol{k_\delta^{-1}}$**) of the complex may approximate the relaxation time. Yet, the relaxation time is shorter than** $\boldsymbol{k_\delta^{-1}}$**, because free A and B are getting depleted over time as a result of their complex formation and therefore the complex formation rate** $\boldsymbol{k_a[A(t) - C(t)][B(t) - C(t)]}$ **in Eq. (1) continues to decline towards quicker relaxation of the complex level. This free-molecule depletion effect to shorten the relaxation time is roughly proportional to the free molecule concentration itself** (Text S1). Hence, the relaxation time takes a decreasing function of the free molecule concentration, consistent with the above observation ⋯"

The Reviewer made the following comment:

> *"3. Page 6, Line 151, Page 15, Line 460. Recent studies have shown that using the tQSSA (more specifically, Eq. (5)) is more accurate than the MM rate law in predicting drug clearance and drug-drug interaction mediated by enzyme induction (10.1111/cts.12804, 10.1002/cpt.2824)."*

We appreciate Reviewer's valuable point and have cited these two references in Section *Parameter estimation* in the revised main text.

> *"4. Figure 2 (d). The explanation for Figure 2 (d) in the main text is insufficient. Moreover, Figure 2 (d) seems unnecessary to understand the analytical response time calculation. It can be moved to the Supporting Information."*

Following Reviewer's suggestion, we have moved Fig. 2(d) to Fig. S6.

> *"5. Figure 4 (a), (b). Figure 4 (a) and (b) do not intuitively illustrate the comparison between the relative errors of parameter estimation based on QSSA and ETS. We suggest a heatmap or box plot for the relative error comparison."*

We are thankful for Reviewer's excellent suggestion. For more intuitive illustration of the relative errors of the parameters estimated by the ETS and (t)QSSA, we have replaced Figs. 4(a) and 4(b) by their scatter plots, as follows:

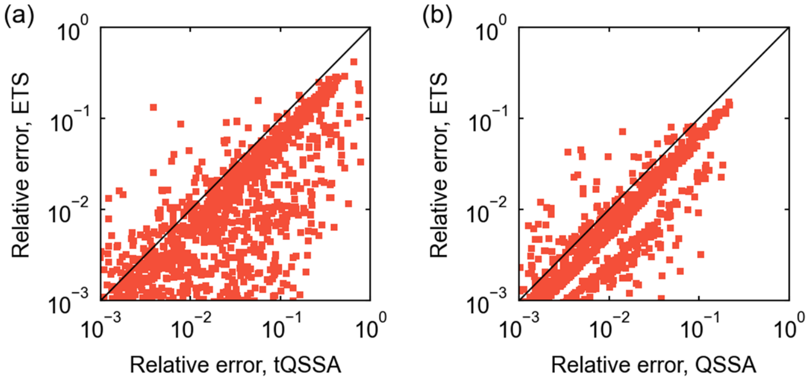

Revised Figs. 4(a) and 4(b). Parameter estimation for protein–protein and TF–DNA interaction models. (a) The scatter plot of the relative errors of the tQSSA- and ETS-estimated K values for a protein–protein interaction model. (b) The scatter plot of the relative errors of the QSSA- and ETS-estimated K values for a TF–DNA interaction model. In (a) and (b), a diagonal line corresponds to the cases where the two estimates have the same relative errors.

In addition, we have accordingly revised the following paragraph in Section *Parameter estimation* of the main text (the revised part in bold):

> "In the case of protein–protein interactions, Fig. 4(a) reveals that the ETS tends to improve the parameter estimation over the tQSSA, with more accurately estimated K: **most of K values (89.4%) estimated by the ETS show smaller relative errors than the tQSSA-based estimates and their 69.3% even show relative errors less than half the tQSSA's.** In the case of TF–DNA interactions, the ETS still offers an improvement in the estimation of K [Fig. 4(b)]: **most of the ETS-estimated K values (90.3%) show smaller relative errors than the QSSA-estimated ones and their 51.8% even show relative errors less than half the QSSA's.**"

The Reviewer made the following comment:

> *"6. In the 'Rhythmic degradation of circadian proteins' section, although the authors referred to Text S11 when they introduce the kinetic model (Line 368–369), it would be better to write the main model equations in the main text to understand the section."*

Following Reviewer's excellent suggestion, we have explicitly described our model equations in the revised main text, as follows:

> "The model comprises the following equations:
>
> $$\frac{\mathrm{d}A_0(t)}{\mathrm{d}t} = g(t) - a_0 A_0(t), \quad (10)$$
>
> $$\frac{\mathrm{d}A_i(t)}{\mathrm{d}t} = a_{i-1} A_{i-1}(t) - a_i A_i(t), \quad (11)$$
>
> where $A_0(t)$ and $A_i(t)$ represent the concentrations of the unmodified and i-th modified proteins, respectively ($i = 1, 2, \cdots, n$ and n is the total number of the PTMs with $n \geq 1$), $g(t)$ is the protein production rate through mRNA expression and translation, a_i denotes the protein's $(i + 1)$-th modification rate ($i = 0, 1, \cdots, n - 1$), and a_n denotes the turnover rate of the n-th modified protein."

The Reviewer made the following comment:

> *"7. Page 18, Line 531-534. The generalization of Eq. (7) to when the number of DNA binding sites is an arbitrary natural number, called the stochastic QSSA, has been derived in previous studies (10.1371/journal.pcbi.1005571, 10.1371/journal.pcbi.1008952). In addition, by comparing the stochastic QSSA with the deterministic QSSAs (i.e., tQSSA and sQSSA), studies have derived the validity conditions for using the deterministic QSSAs in stochastic models, which are generally stricter than those in deterministic models (10.1016/j.bpj.2014.06.012, 10.1371/journal.pcbi.1008952). Extending these studies using ETS would be an interesting candidate for the discussed future work."*

We are thankful for Reviewer's valuable comment. According to this comment, we have added the following sentences and references to the revised main text [the condition $VK\Delta_{\mathrm{tQ}}(t) \gg 1$ noted below, which is a sufficient condition for the deterministic scheme, comes from our interpretation of Eq. (7) in the Reviewer-mentioned paper 10.1371/journal.pcbi.1008952]:

> (Section *Theory development*) "In fact, $C_{\mathrm{TFQ}}(t)$ in Eq. (7) corresponds to a special case of the previously-studied, *stochastic quasi-steady state approximation* (stochastic QSSA) [33,34] for arbitrary molecular copy numbers such as for multiple DNA binding sites. Of note, the stochastic QSSA becomes close to the tQSSA as its deterministic version, if $VK\Delta_{\mathrm{tQ}}(t) \gg 1$ [34]."

> (Section *Discussion*) "Lastly, comprehensive consideration of stochastic fluctuations in molecular binding events [32,57,58] beyond the TF–DNA interactions in this study would be a fruitful endeavor for more complete development of our theory, through possible extension of the existing stochastic QSSA [33,34]."

(Section *References*)

"33. J. K. Kim and E. D. Sontag, *Reduction of Multiscale Stochastic Biochemical Reaction Networks Using Exact Moment Derivation*, PLOS Comput. Biol. **13**, e1005571 (2017).

34. Y. M. Song, H. Hong, and J. K. Kim, *Universally Valid Reduction of Multiscale Stochastic Biochemical Systems Using Simple Non-Elementary Propensities*, PLOS Comput. Biol. **17**, e1008952 (2021)."

Additionally, we have revised Text S4 in a similar way.

"8. Text S1 and S4. The main theoretical results of the paper are presented in these sections. These sections, or at least the summary of these sections, need to be provided in the main text."

We appreciate Reviewer's constructive suggestion. Because the entire inclusion of Texts S1 and S4 in the main text may lead some readers to feel mathematically overloaded with rather lower readability, we have instead provided the summary of Texts S1 and S4 in the revised main text, as follows (Section *Theory development*; the revised part in bold here):

"Still, both the tQSSA and sQSSA stand on the assumption that $C(t)$ approaches the quasi-steady state fast enough each time before the marked temporal change of $A(t)$ or $B(t)$. We now relax this quasi-steady state assumption and generalize the approximation of $C(t)$ to the case of time-varying $A(t)$ and $B(t)$, as the main objective of this study.

Suppose that $C(t)$ may not necessarily approach the quasi-steady state each time but stays within some distance from it. **As detailed in Text S1, we linearize the right-hand side of Eq. (1) around $C(t) - C_{\mathrm{tQ}}(t)$ and estimate $C(t)$'s solution as the time integral of $C_{\mathrm{tQ}}(t')$ (where $t' \le t$) with an exponential kernel-like function. The Taylor expansion of $C_{\mathrm{tQ}}(t')$ by $t - t'$ is incorporated into this integral and then its form offers the following approximant for $C(t)$:**

$$C_{\gamma}(t) \equiv \min\left\{C_{\mathrm{tQ}}\left\{t - \left[k_{\delta}\Delta_{\mathrm{tQ}}(t)\right]^{-1}\right\}, A(t), B(t)\right\}. \qquad (6)"$$

"Still, the use of $C_{\mathrm{TFQ}}(t)$ stands on the quasi-steady state assumption. We relax this assumption and generalize the approximation of $C_{\mathrm{TF}}(t)$ to the case of time-varying TF concentration. **In a similar way to obtain $\boldsymbol{C_\gamma(t)}$ in Eq. (6),** we propose the following approximant for $C_{\mathrm{TF}}(t)$ (Text S4):

$$C_{\mathrm{TF}\gamma}(t) \equiv C_{\mathrm{TFQ}}\left[t - \frac{k_\delta^{-1}K}{K + A_{\mathrm{TF}}(t)}\right]. \quad (8)$$"

The Reviewer asked the following question:

"9. Text S5. Eq. (S29) was derived from Eq. (S7) using Eq (S14), which was derived under the assumption of Eq. (S7). Is Eq. (S29) equivalent to Eq. (S7)?"

Eq. (S29) is a "self-consistency" condition with Eq. (S14). Because Eq. (S14) itself is an approximation, Eq. (S29) is not strictly equivalent to Eq. (S7), but can be viewed as approximately equivalent to Eq. (S7).

"10. Text S7. The calculation of the phase differences between the approximations (i.e., ETS, tQSSA, and sQSSA) and the actual time course of C is unclear."

We are thankful for this careful comment. Accordingly, we have cited Text S12 in the revised Texts 7 and 8 for the phase difference calculation. Text S12 explains the details of our phase difference calculation, as follows:

"A phase difference between two periodic time-series was calculated by maximizing their cross-correlation with a varying displacement of one series relative to the other [S21]. For the cross-correlation calculation, the time average of each series was shifted to zero and ten duplicates of a single time period ($10 \times T$) was used. The cross-correlation was obtained with signal.correlate in SciPy v1.1.0 or v1.3.1 (mode = 'same' and method = 'fft')."

The Reviewer made the following comment:

"11. Text S5. The derivation of "Delta_{tQ}^{-2} Delta'_{tQ} << 1" is unclear."

Eq. (S11) is a condition that $\Delta_{\mathrm{tQ}}(\tau') \approx \Delta_{\mathrm{tQ}}(\tau)$ for τ' in the range $\tau - \Delta_{\mathrm{tQ}}^{-1}(\tau) \lesssim \tau' \leq \tau$. By applying an expansion $\Delta_{\mathrm{tQ}}(\tau') \approx \Delta_{\mathrm{tQ}}(\tau) + (\tau' - \tau)\Delta'_{\mathrm{tQ}}(\tau)$ to Eq. (S11), we obtain $\left|(\tau' - \tau)\Delta'_{\mathrm{tQ}}(\tau)\right| \ll \Delta_{\mathrm{tQ}}(\tau)$ for τ' in the range $\tau - \Delta_{\mathrm{tQ}}^{-1}(\tau) \lesssim \tau' \leq \tau$ and therefore $\Delta_{\mathrm{tQ}}^{-2}(\tau)\left|\Delta'_{\mathrm{tQ}}(\tau)\right| \ll 1$. We have added these details to the revised Text S5.

"12. In Text S12, add subsection titles to improve readability."

We agree to Reviewer's valuable suggestion. Following this suggestion, we have added subheadings to the revised Text S12 for better readability.

Response to Reviewer #2

First of all, we are very thankful for Reviewer's careful comment. The Reviewer kindly appreciated our work as *"The manuscript describes a novel multiscale approximation ⋯ Overall, the paper is well written ⋯ I did not find any technical errors. As such, I do not have any major concerns."*

On the other hand, the Reviewer pointed out the following aspect: *"Minor comment: The description/interpretation of various quasi-steady-state approximations is rather narrow/simplistic. While the description provided in "Theory overview" section is in principle okay, it is far from comprehensive. In particular, it misses out stochastic quasi-steady-state approximations and how they relate to deterministic ones."*

In response to the above valuable comment, we stress that Section *Theory overview* is presented under Section *Results*. In this section, we included only the existing approaches that are directly relevant to our results, because the comprehensive review of all the approaches could rather compromise the focus of our study. To better reflect the nature of this section, we have now changed the section title *Theory overview* to *Theory development* in the revised main text.

Nevertheless, we completely agree to Reviewer's view that this section shall include the discussion of the stochastic quasi-steady state approximation (stochastic QSSA). Therefore, we have added the following sentences and references to our revised main text [the condition $VK\Delta_{\mathrm{tQ}}(t) \gg 1$ noted below, which is a sufficient condition for the deterministic scheme (tQSSA), comes from our interpretation of Eq. (7) in Ref. [34] of the revised manuscript]:

> (Section *Theory development*) "In fact, $C_{\mathrm{TFQ}}(t)$ in Eq. (7) corresponds to a special case of the previously-studied, *stochastic quasi-steady state approximation* (stochastic QSSA) [33,34] for arbitrary molecular copy numbers such as for multiple DNA binding sites. Of note, the stochastic QSSA becomes close to the tQSSA as its deterministic version, if $VK\Delta_{\mathrm{tQ}}(t) \gg 1$ [34]."
>
> (Section *Discussion*) "Lastly, comprehensive consideration of stochastic fluctuations in molecular binding events [32,57,58] beyond the TF–DNA interactions in this study would be a fruitful endeavor for more complete

development of our theory, through possible extension of the existing stochastic QSSA [33,34]."

(Section *References*)

"33. J. K. Kim and E. D. Sontag, *Reduction of Multiscale Stochastic Biochemical Reaction Networks Using Exact Moment Derivation*, PLOS Comput. Biol. **13**, e1005571 (2017).

34. Y. M. Song, H. Hong, and J. K. Kim, *Universally Valid Reduction of Multiscale Stochastic Biochemical Systems Using Simple Non-Elementary Propensities*, PLOS Comput. Biol. **17**, e1008952 (2021)."

Additionally, we have revised Text S4 in a similar way.

Response to Reviewer #3

We are thankful for Reviewer's careful comments. Here, we offer a list of the changes made in the manuscript in response to Reviewer's comments.

> *"1. One of the main problems with this work is that the authors are not familiar with modern principles of theoretical enzymology. As a result of this, they introduce a number of ideas, which are fundamentally correct. For example, they assume that the quasi-steady-state approximations results from the rapid equilibrium of the complex concentration. This is fundamentally incorrect. The quasi-steady-state approximation never assumes equilibrium. It naturally arises as a result of the existence of natural scaling, which separates the reaction in two regimes: a fast regime, and a slow one."*

We understand that the quasi-steady state approximation in chemical reaction kinetics is an outcome of the separation of fast and slow time-scales, as already stressed in Line 504 of our original manuscript as "The quasi-steady state assumption involves the approximation by time-scale separation ⋯": the intermediate complex reaches its quasi-steady state rapidly, compared to relatively slow changes in reactant concentrations.

Yet, the word "equilibrium" in our manuscript was loosely adopted to indicate the quasi-steady state, as the formation and decay rates of the complex become balanced at each quasi-steady state. In other words, the "equilibrium" here is neither a thermodynamically-equilibrated state nor a permanent steady state. This word was just chosen for an intuitive delivery of the quasi-steady state assumption to a broad readership, given the common use of this word in non-technical contexts.

We also kindly note that the other two Reviewers did not raise any concerns about the use of "equilibrium" in our manuscript, indicating little chance of its misinterpretation within the context of our manuscript.

Nevertheless, we fully agree to Reviewer's above view in favor of technically more precise words. **Therefore, throughout the revised manuscript, we have mostly replaced the word "equilibrium" by the word "quasi-steady state". For only a few places where the use of "equilibrium" is still intuitively appealing with little chance of misinterpretation, we leave that word as it is, or have added "(quasi-steady state)" next to the word.**

"2. The authors claim the superiority of the total quasi-steady-state approximation over the standard quasi-steady-state approximation. The foundations of this superiority are not set in stone, but rather moving sands. Supporters of the total quasi-steady-state approximation select parameters and made numerical simulations, where the approximation shows improvements with respect to the standard quasi-steady-state approximation. Also the total quasi-steady-state approximation has more parameters, which make it much more difficult to implement and uniquely identify parameters. There has not been a systematic study to demonstrating than one approximation is better than the other."

We understand Reviewer's comment. However, we kindly point out that **the total quasi-steady state approximation (tQSSA) for the concentration of complex has only a single parameter (Michaelis constant) and this case is the same for the standard quasi-steady state approximation (sQSSA).** This point is evident from the tQSSA's following form:

$$C_{\mathrm{tQ}}(t) = \frac{1}{2}\left\{K + A(t) + B(t) - \sqrt{[K + A(t) + B(t)]^2 - 4A(t)B(t)}\right\},$$

where $A(t)$ and $B(t)$ denote the total concentrations of A and B molecules, respectively, $C_{\mathrm{tQ}}(t)$ denotes the tQSSA-based concentration of AB complex, and K is the Michaelis constant (commonly written as K_{M}).

The above formula clarifies the following three points: (*i*) $C_{\mathrm{tQ}}(t)$ from the tQSSA involves only a single parameter K as in the case of the sQSSA; (*ii*) the form of $C_{\mathrm{tQ}}(t)$ does not look as elegant as that of the sQSSA, but it is just as easy to implement in a computer program; (*iii*) $C_{\mathrm{tQ}}(t)$ is a differentiable and monotonically-decreasing function of K, as evident from the partial derivative of $C_{\mathrm{tQ}}(t)$ by K.

Based on (*i*) to (*iii*) above, we are afraid that we do not agree to Reviewer's viewpoint that *"the total quasi-steady-state approximation has more parameters, which make it much more difficult to implement and uniquely identify parameters"*.

In contrast to Reviewer's comment *"The foundations of this* [tQSSA's] *superiority are not set in stone, but rather moving sands"*, **the validity conditions for the tQSSA have been systematically derived in previous studies** [e.g., *Bull. Math. Biol.* **65**, 1111 (2003); *Math. Biosci.* **325**, 108339 (2020)] **and the higher accuracy of the tQSSA**

than the sQSSA's is widely accepted among researchers, as recently reviewed in this journal, *PLOS Computational Biology* itself [*PLOS Comput. Biol.* **16**, e1008258 (2020)]. Even our own analysis supports the better performance of the tQSSA, as demonstrated by Figs. S2(b), S3(e), and S8 in our revised manuscript.

Taken together, Reviewer's concern seems to be directed towards the entire body of the tQSSA-preferring studies that are the majority in this field, rather than only limited to our study. Therefore, **it will be really grateful if the Reviewer kindly recommends some specific references against this major view**, because we have not yet been able to find such references despite our efforts.

> *"3. The conditions introduced for the validity of the standard quasi-steady-state approximation - originally derived by Lee Segel - are outdated. There has been much more rigorous estimates calculated, where there is no dependency between the substrate and enzyme concentration."*

To follow Reviewer's valuable suggestion, we have cited more recent studies about the validity conditions of the sQSSA as Refs. [15–17] in our revised manuscript [*FEBS J.* **281**, 464 (2014); *AIMS Math.* **6**, 6781 (2021); *Math. Biosci.* **350**, 108870 (2022)].

Still, to our knowledge, the sQSSA requires the inequality between substrate and enzyme levels for its validity. Therefore, regarding the above comment *"⋯ there is no dependency between the substrate and enzyme concentration"*, it will be really grateful if the Reviewer recommends specific references for this comment.

> *"4. This reviewer doesn't understand how Eq (1) was derived, after reading Text S1. There seems to be a fundamental problem with the derivation and assumptions. Maybe I am wrong, but I couldn't follow the derivations as it is unclear how the authors have derived the total A, A(t), and total B, B(t), concentrations. They give the impression that the free A and B concentration are equal to A(t)-C(t) and B(t)-C(t). However, the reaction schemes in Figure 1 are both open. As a result of this, there is no conserve quantities. The application of the total concentrations generally requires to work with conserved reactions. As a result of this, it is unclear if the authors are applying the total-quasi-steady-state approximation well."*

In our study, the temporal profiles of $A(t)$ and $B(t)$ are allowed to be very generic and they don't have to be constant. **By the definition of $A(t)$ as the "total" concentration of A, $A(t)$ is the sum of its free and bound molecules** in any cases [generally, there is no other form of A; for example, a chemical-reaction product from the chemical conversion of the bound form of A is simply another molecule chemically-distinguished from A and thus does not contribute to $A(t)$]**.** Therefore, it is straightforward that **the concentration of free A equals $A(t) - C(t)$**, where $C(t)$ is the concentration of AB complex. In the same way, **the concentration of free B equals $B(t) - C(t)$**. From the law of mass action, Eq. (1) in the manuscript is straightforward to obtain in the following form:

$$\frac{\mathrm{d}C(t)}{\mathrm{d}t} = k_{\mathrm{a}}[A(t) - C(t)][B(t) - C(t)] - k_{\delta}C(t).$$

Importantly, for the sake of generality, we define k_{δ} as the sum of all kinds of rates to decrease $C(t)$. In other words, **k_{δ} is not limited to AB's dissociation but also embraces all other events to lower the level of AB in the system. Therefore, it is applicable for both closed and open reaction schemes. Specifically, $k_{\delta} \equiv k_{\mathrm{d}} + r_{\mathrm{c}} + k_{\mathrm{loc}} + k_{\mathrm{dlt}}$ where k_{d}, k_{loc}, and k_{dlt} stand for the dissociation, translocation, and dilution rates of AB, respectively, and r_{c} for the chemical conversion (e.g., chemical reaction or degradation) or translocation rate of A or B upon the formation of AB.**

Using this k_{δ}, together with k_{a} in the above equation, we define $K \equiv k_{\delta}/k_{\mathrm{a}}$ and plug it in the tQSSA of the complex concentration, $C_{\mathrm{tQ}}(t) = (1/2) \times \left\{K + A(t) + B(t) - \sqrt{[K + A(t) + B(t)]^2 - 4A(t)B(t)}\right\}$. **This form of the tQSSA is the solution under the quasi-steady state assumption**, **because the time derivative $C'(t)$ in the above equation becomes zero when $C(t) = C_{\mathrm{tQ}}(t)$** (replacing $C(t)$ in the right-hand side of the above equation by the formula of $C_{\mathrm{tQ}}(t)$ easily proves this fact and no alternative tQSSA solution exists due to the condition that $C(t)$ should be smaller than each $A(t)$ or $B(t)$. The full details are provided in the first page of Text S1).

We kindly hope that all this procedure is now clear, as the other two Reviewers did not raise any technical concerns about the procedure.

Thanks to Reviewer's comment, we have clarified our points in the revised main text and Text S1. For example, the description of Eq. (1) in the main text has been revised as follows (the revised part in bold):

> "Consider two different molecules A and B that bind to each other and form complex AB, as illustrated in Fig. 1(a) ⋯ The concentration of the complex AB at time t, denoted by $C(t)$, changes over time as in the following equation **from the mass-action law**:
>
> $$\frac{\mathrm{d}C(t)}{\mathrm{d}t} = k_{\mathrm{a}}[A(t) - C(t)][B(t) - C(t)] - k_{\delta}C(t). \quad (1)$$
>
> Here, $A(t)$ and $B(t)$ denote the total concentrations of A and B, respectively, and hence **$A(t) - C(t)$ and $B(t) - C(t)$ are the concentrations of free A and B**. The temporal profiles of $A(t)$ and $B(t)$ are allowed to be very generic, e.g., even with their own feedback effects as addressed later. k_{a} denotes the association rate of free A and B. k_{δ} is the effective "decay" rate of AB with $k_{\delta} \equiv k_{\mathrm{d}} + r_{\mathrm{c}} + k_{\mathrm{loc}} + k_{\mathrm{dlt}}$ where k_{d}, k_{loc}, and k_{dlt} stand for the dissociation, translocation, and dilution rates of AB, respectively, and r_{c} for the chemical conversion or translocation rate of A or B upon the formation of AB. **In other words, for the sake of generality, k_{δ} is not limited to a dissociation event but encompasses all rate events to lower the level of AB** [Fig. 1(a)]."

The Reviewer made the following two comments:

> *"6. The time-delay scheme solution is very similar - structurally - to the quasi-steady-state approximations rate laws. It remains unclear the precise parameter domains, where the time-delay scheme rate law is valid. As an approximation, it must have some limitation and range of validity. The paper doesn't seem to the present one, or has a serious discussion about the validity of the new time-delay approximation.*
>
> …
>
> *8. It is also unclear if it is fair to compare a quasi-steady-state approximations with the time-delay approximation derived in this paper. By nature, they seems to be very different approximations, which will be valid under a different set of experimental conditions."*

We here address these two comments together. Our time-delay scheme, termed the ETS, is the "correction" of the quasi-steady state approximation and can be applied to a wider range of conditions, e.g., that may involve actively time-varying molecular concentrations and do not necessarily adhere to the quasi-steady state assumption. Because the existing quasi-steady state approximations have previously been even applied to such actively time-varying cases, **it is fair to compare the ETS to the quasi-steady state approximations** in these time-varying cases, as demonstrated through various biochemical examples in our study (main text and Texts S6–S11).

About the parameter conditions for the validity of the ETS in Reviewer's question, their systematic derivation is provided in Text S5 and this Text S5 is cited at the end of Section *Theory development* in the revised main text. Briefly, Eqs. (S29), (S31), and (S32) in Text S5 are the validity conditions of the ETS. In the case of TF–DNA interactions, they are Eqs. (S35) and (S36), instead.

> *"7. Mathematical modeling with time delay comes with challenges. Delay-differential equations can have an infinity number of solutions. Parameter estimation has the same problem. To cap it all, delay differential equation numerical tools are not widely available, and require substantial expertise to be handled. It is not the typical tool used by a biochemist. This is an issue of major concern about the practical utility of the new approach."*

We appreciate Reviewer's insightful comment. We would like to emphasize that our goal was not to provide a practical recipe, but rather to establish a novel conceptual framework for kinetics research. Nevertheless, implementing our method is not as difficult as imagined, for the following reasons:

About the above comment "Delay-differential equations can have an infinity number of solutions". Because our time-delay scheme (ETS) involves $C_{\mathrm{tQ}}\left\{t-\left[k_\delta \Delta_{\mathrm{tQ}}(t)\right]^{-1}\right\}$ [where $C_{\mathrm{tQ}}(t)$ is the tQSSA of the complex concentration], its value is uniquely determined once $t \geq t_0+\left[k_\delta \Delta_{\mathrm{tQ}}(t)\right]^{-1}$ with t_0 as an initial time point of the system. Notably, $\left[k_\delta \Delta_{\mathrm{tQ}}(t)\right]^{-1}$ is the molecular relaxation time in complex formation, according to Eq. (S12). In other words, the ETS takes an arbitrary value only during a transient period under the relaxation process of the arbitrary initial condition of the complex concentration. This property captures a quite natural phenomenon, given that

a transient behavior of the full exact ODE model (without any approximations such as the ETS) depends on the initial condition of the complex concentration too, before reaching its unique value. It is thus obvious that the other rate laws, such as the tQSSA and sQSSA, are not applicable to this transient period, either. These points are discussed in the last paragraph of Text S1.

In line with the above issue, to our numerical simulation experience, the asymptotic solutions of the delay differential equations with the ETS have always been uniquely determined despite various initial conditions, as long as their exact counterparts with the full ODE models exhibited such behaviors. This insensitivity to initial conditions is noted in Text S12's subsections related to Texts S6–S11.

Still, we agree to Reviewer's view that more systematic investigation would be warranted in the future about the convergence issue of those delay differential equations.

About the above comment "delay differential equation numerical tools are not widely available" . Computing the numerical solutions of delay differential equations can be challenging. Nevertheless, it has been implemented in various programming languages with relevant packages. MALTLAB, which is widely used for modeling and simulation, provides *dde23* and *ddesd* functions for solving delay differential equations. *DelayDiffEq* (a Julia library), *dede* (a R library), and *ddeint* and *jitcdde* (Python libraries) can also be used for delay differential equations. These libraries utilize numerical interpolation methods and libraries for ODE integrators. **They also provide detailed tutorial documents for users.**

MATLAB and Python are widely used in scientific computing. **It won't be difficult for scientists to use the above functions and libraries if they are familiar with MATLAB or Python.**

In our study, **we used *ddeint* to solve delay differential equations because our codes are based on Python** as described in Text S12. These codes are available at GitHub (https://github.com/rokt-lim/Generalized_Michaelis-Menten_rate_law).

To implement the parameter estimation involving time-delay terms, we applied Powell's method with scipy.optimize.minimize in Python. The full details of this parameter estimation are explained in Text S12's subsection titled "Section *Parameter estimation* in the main text".

"9. The effectiveness of the parameter estimation with the new rate law is not robust enough to determine if the new rate law is an impactful contribution to the literature. It is done in comparison with the total quasi-steady-state equation. Comparisons are limited to a restricted set of conditions, and remains unclear if it will be valid under a broader set of parameter domains."

We are thankful for Reviewer's valuable comment. In response to this comment, we have first compared the parameter estimation accuracies of the tQSSA and sQSSA each other, as two quasi-steady state approximations. Panel (a) in the figure below shows that the tQSSA-based parameter estimates are more accurate than the sQSSA-based ones: most of the tQSSA-based estimates (77.4%) exhibit smaller relative errors than the sQSSA-based ones. **The tQSSA's better parameter estimation is also supported by Refs. [12,49] in the revised main text.**

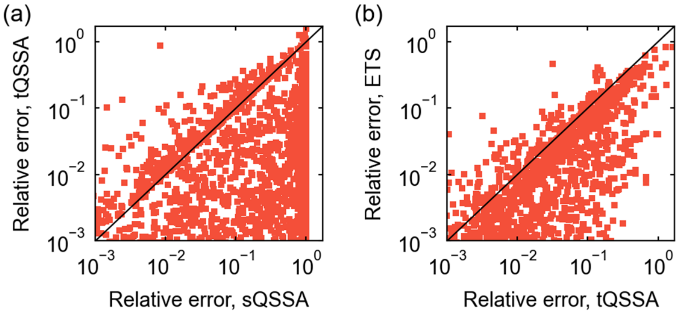

Comparison of parameter estimation accuracies. (a) The scatter plot of the relative errors of the sQSSA- and tQSSA-estimated K values. (b) The scatter plot of the relative errors of the tQSSA- and ETS-estimated K values. In (a) and (b), a diagonal line corresponds to the cases where the two estimates have the same relative errors. For more details, refer to Text S12.

In this respect, we only focus on the comparison between the ETS (our time-delay scheme) and the tQSSA, without the further use of the sQSSA that is less accurate than the tQSSA. Panel (b) in the figure above shows that the ETS-based parameter estimates are even more accurate than the tQSSA-based ones: most of the ETS-based estimates (89.4%) exhibit smaller relative errors than the tQSSA-based ones and their 69.3% even show relative errors less than half the tQSSA's.

All these results are now presented in new Figs. 4(a) and S8 and their descriptions in the revised manuscript. About Reviewer's comment *"Comparisons*

are limited to a restricted set of conditions, and remains unclear if it will be valid under a broader set of parameter domains", we kindly stress that **our parameter space covers the almost maximum extent of physiologically-relevant ranges, as their references are thoroughly cited in Table S3. Yet, in order to address Reviewer's concern, we have even extended this parameter space** (e.g., to half the minimum and twice the maximum range of k_δ), **but have not found much different results from the above:** (*i*) still, the tQSSA-based parameter estimates remain more accurate than the sQSSA-based ones, as most of the former ones (78.5%) exhibit smaller relative errors than the latter; (*ii*) also, the ETS-based parameter estimates remain more accurate than the tQSSA-based ones, as most of the former ones (89.4%) exhibit smaller relative errors than the latter.
