# Supplementary material for: Generalized Michaelis–Menten rate law with time-varying molecular concentrations
Source: PLoS Comput Biol. 2023 Dec 11;19(12):e1011711. doi: 10.1371/journal.pcbi.1011711 (PMC10735182; doi:10.1371/journal.pcbi.1011711)
Supplement: Attachment — Submitted filename: Response_to_Reviewers_Kim.pdf [file pcbi.1011711.s004.pdf]

Response to Reviewer #1

First of all, we express our gratitude for Reviewer's kind recommendation that *"I appreciate the authors' efforts in addressing my previous comments. They have resolved all the concerns I raised, resulting in a notable enhancement in the manuscript's clarity. I recommend the manuscript for publication."*

The Reviewer made the following last comment: *"Equations (S10) to (S12): The authors derive (S12) from (S10) by setting the integral range from -∞ to τ and omitting the term containing ¯C(τ_0). This derivation appears somewhat unclear, making it difficult to ascertain the validity of this step. I kindly request the authors to provide additional details regarding this derivation process and the underlying assumptions made."*

We appreciate Reviewer's constructive suggestion. Accordingly, we have provided additional details in the revised Text S1, as follows (the revised part in bold):

> [Text S1, below Eq. (S11)] "**We then apply Eq. (S11) to Eq. (S10) and notice that $\int_{\tau'}^{\tau} \Delta_{\mathrm{tQ}}(\tau'')\mathrm{d}\tau'' \approx (\tau - \tau')\Delta_{\mathrm{tQ}}(\tau)$ and $\int_{\tau_0}^{\tau} \Delta_{\mathrm{tQ}}(\tau')\,\mathrm{d}\tau' \approx (\tau - \tau_0)\Delta_{\mathrm{tQ}}(\tau_0)$ for $\tau' \gtrsim \tau - \Delta_{\mathrm{tQ}}^{-1}(\tau)$ and $\tau \lesssim \tau_0 + \Delta_{\mathrm{tQ}}^{-1}(\tau_0)$, respectively. Subsequently, $\exp\left[-\int_{\tau'}^{\tau} \Delta_{\mathrm{tQ}}(\tau'')\mathrm{d}\tau''\right] = \exp\left[-\int_{\tau-\Delta_{\mathrm{tQ}}^{-1}(\tau)}^{\tau} \Delta_{\mathrm{tQ}}(\tau'')\mathrm{d}\tau'' - \int_{\tau'}^{\tau-\Delta_{\mathrm{tQ}}^{-1}(\tau)} \Delta_{\mathrm{tQ}}(\tau'')\mathrm{d}\tau''\right] \approx \exp\left[-1 - \int_{\tau'}^{\tau-\Delta_{\mathrm{tQ}}^{-1}(\tau)} \Delta_{\mathrm{tQ}}(\tau'')\mathrm{d}\tau''\right]$, $\exp\left[-\int_{\tau_0}^{\tau} \Delta_{\mathrm{tQ}}(\tau')\,\mathrm{d}\tau'\right] = \exp\left[-\int_{\tau_0}^{\tau_0+\Delta_{\mathrm{tQ}}^{-1}(\tau_0)} \Delta_{\mathrm{tQ}}(\tau')\,\mathrm{d}\tau' - \int_{\tau_0+\Delta_{\mathrm{tQ}}^{-1}(\tau_0)}^{\tau} \Delta_{\mathrm{tQ}}(\tau')\,\mathrm{d}\tau'\right] \approx \exp\left[-1 - \int_{\tau_0+\Delta_{\mathrm{tQ}}^{-1}(\tau_0)}^{\tau} \Delta_{\mathrm{tQ}}(\tau')\,\mathrm{d}\tau'\right]$, and the former and latter values become negligible for $\tau' \ll \tau - \Delta_{\mathrm{tQ}}^{-1}(\tau)$ and $\tau \gg \tau_0 + \Delta_{\mathrm{tQ}}^{-1}(\tau_0)$, respectively. Also, $\exp\left[-(\tau - \tau')\Delta_{\mathrm{tQ}}(\tau)\right]$ becomes negligible for $\tau' \ll \tau - \Delta_{\mathrm{tQ}}^{-1}(\tau)$. Therefore, combined with Eq. (S11), Eq. (S10) for $\tau \gg \tau_0 + \Delta_{\mathrm{tQ}}^{-1}(\tau_0)$ is approximated as**
>
> $$\bar{C}(\tau) \approx \Delta_{\mathrm{tQ}}(\tau) \int_{-\infty}^{\tau} \bar{C}_{\mathrm{tQ}}(\tau') e^{-(\tau-\tau')\Delta_{\mathrm{tQ}}(\tau)} \mathrm{d}\tau', \qquad \text{(S12)}$$
>
> **where the right-hand side is not sensitive to the specific lower limit of τ' for the integral as long as this lower limit is $\ll \tau - \Delta_{\mathrm{tQ}}^{-1}(\tau)$.**"

To complement the above, the description before Eq. (S12) ensures that the integral in Eq. (S12) is not sensitive to the specific lower bound of τ' as long as this lower bound is $\ll \tau - \Delta_{\mathrm{tQ}}^{-1}(\tau)$, and we therefore chose $-\infty$ for analytical convenience towards Eq. (S13).

Response to Reviewer #2

First of all, we are very thankful for Reviewer's comment. The Reviewer kindly appreciated our previous response as *"My concerns have been sufficiently addressed"*.

The Reviewer suggested *"I would like to bring to the author's attention the works of Tom Kurtz and colleagues on multiscale approximation methods that are also used to perform quasi-steady-state approximations.*

1. Separation of time-scales and model reduction for stochastic reaction networks. Hye-Won Kang, and Tom Kurtz. Annals of Applied Probability.

2. Asymptotic analysis of multiscale approximations to reaction networks. Ball, Kurtz, Popovic, Rempala. Annals of Applied Probability.

3. Quasi-Steady-State Approximations Derived from the Stochastic Model of Enzyme Kinetics. Kang, KhudaBukhsh, Koeppl, and Rempala. Bulletin of Mathematical Biology."

According to this valuable comment, we have added the following references to our revised main text and cited them in Section *Discussion*:

(Section *References*)

"56. K. Ball, T. G. Kurtz, L. Popovic, and G. Rempala, *Asymptotic Analysis of Multiscale Approximations to Reaction Networks*, Ann. Appl. Probab. **16**, 1925–1961 (2006).

57. H.-W. Kang and T. G. Kurtz, *Separation of Time-Scales and Model Reduction for Stochastic Reaction Networks*, Ann. Appl. Probab. **23**, 529–583 (2013)."

Response to Reviewer #3

We are very thankful for Reviewer's constructive comments. Here, we offer a list of the changes made in the manuscript in response to Reviewer's comments.

> *"*** Your response to Comment 2 – Part I*
> *Yes, you are right that the expression for the complex concentration has only a single parameter in the tQSSA. However, the same can be said for the standard QSSA approximation. The fundamental problem is two-fold: (i) In the laboratory, we can rarely observed the complex concentration; it is a short-lived chemical intermediate, particularly for steady-state kinetic experiments. My lab would like to measure complex intermediate concentrations, but we can only do this under conditions, where the complex is not anymore a short-lived intermediate, but it is the core of the reaction. (ii) The complexity of the tQSSA lies in the total substrate concentration experiments, where there are more parameters. Additionally, it requires to measure in the laboratory the total substrate, which is not a directly observable chemical species as it requires to measure both the free substrate and intermediate complex concentration."*

Although it is rather challenging to quantify transient metabolite–enzyme complex in a laboratory, nuclear magnetic resonance (NMR) spectroscopy can serve this purpose and provide quantitative information of both the free and complex forms for the "total" concentration that the Reviewer asked how to measure. In addition, other methods are also available for specific cases. For example, if the metabolite or enzyme is intrinsically fluorescent or labelable with a fluorophore, fluorescence spectroscopy would be a choice to study their interaction.

More fundamentally, as reviewed in Section *Introduction* of our original manuscript, the tQSSA improves the modeling of protein–protein interactions compared to the sQSSA, while their performances are similar to each other in the case of metabolic reactions because the metabolite levels far exceed the enzyme levels and thus the sQSSA is still valid there. In other words, the experiments with protein–protein interactions would be better for testing the tQSSA than those with metabolic reactions. To quantify the protein complex and the total proteins for such test, one can employ mass spectrometry (MS)-based proteomics coupled with co-immunoprecipitation (Co-

IP), densitometry with western blotting, or enzyme-linked immunosorbent assay (ELISA). Thanks to Reviewer's valuable comment, we have added these experimental methods to the main text of our revised manuscript, as follows (the revised part in bold):

> (Section *Discussion*) "This validation [of the ETS] could involve the measurement of the time-series of molecular complex concentrations, **such as by mass spectrometry-based proteomics with co-immunoprecipitation, densitometry with western blotting, and enzyme-linked immunosorbent assay in the case of protein complex quantification.**"

The Reviewer made another comment:

> *"Your repression to data in the supplementary material shows a weak fitting overall to the tQSSA at least much more weaker of what we tend to see in the enzyme kinetics literature. This is typical for complex systems, like the tQSSA expressions."*

The whole point of our manuscript is that **the tQSSA can fail for actively time-varying molecular concentrations** (like transiently-induced or circadian-controlled protein levels) and thus we propose a new method termed the ETS, as the generalization of the tQSSA for such time-varying cases. Therefore, **the relatively poor performance of the tQSSA in those cases is readily expected** and this is exactly what the ETS can resolve, as demonstrated through the consistently higher performance of the ETS than the tQSSA's across numerous biomolecular systems in our manuscript.

> *"*** Your response to Comment 2 - Part II*
> *Your understanding of the validity of the conditions for the tQSSA is not correct. You are reading studies which are using heuristic approaches to derive the equations for the validity of the tQSSA. These approaches and their numerical solutions provide sufficient conditions for the validity of the tQSSA, but not necessary conditions, which have been proven mathematically. As such, most of the conditions published do not guarantee the validity of the approximations. The necessary conditions are much stronger. Let me bring to your attention paper 17 that you cited. In this manuscript, the necessary condition for the validity of the tQSSA is (K e0)/(Km+e0)^2 << 1. The analysis*

of 17 shows that tQSSA is not universally valid, as claimed by most, but only on a limiting case.

Of course, it might be possible that the reference 17 is not correct. However, the analysis in 17 seems to be more rigorous to me."

Thanks to Reviewer's valuable comment, we have cited Ref. [17] in one more place (Line 70) in Section *Introduction* of our revised manuscript, where the tQSSA is explained.

On the other hand, as we have already pointed out above, it is readily expected that the tQSSA does not work well for actively time-varying molecular concentrations. Indeed, even the validity condition from Ref. [17] in Reviewer's comment suggests that large K, which leads to rapid substrate depletion over time through product formation, would invalidate the tQSSA. To our knowledge, no researcher in this field suggests the "universal" validity of the tQSSA, because the tQSSA is only the exact solution at the steady state, but not for every case. If the system severely deviates from the steady states, the validity of the tQSSA can be questioned, as a part of the main point of our manuscript.

Despite this limitation of the tQSSA, it still performs better than the sQSSA because the sQSSA is even inaccurate at the steady state unlike the tQSSA's exactness there. The details of this last point were kindly provided in our response to Reviewer's comments in the previous round.

*"*** Your response to Comment 3*

I am glad to hear that you found the references very useful. Regarding the references showing that the sQSSA is not dependent on the strate and enzyme ratio, 17 shows both that the necessary conditions for the validity of sQSSA and tQSSA are not dependent on the substrate to enzyme ratio. The abstract says "we obtain local conditions for the accuracy of standard or total quasi-steady-state. Perhaps surprisingly, our conditions do not involve initial substrate.". In my repsonse above, I provided the condition listed for the tQSSA, which is not dependent on the substrate concentration. If you read 17, or Reich and Selkov [FEBS Lett., 40 (Suppl. 1) (1974), pp. S119-S127] and justified by Palsson and Lightfoot [J. Theoret. Biol., 111 (1984), pp. 273-302],

you will see that the necessary conditions for the validity of the sQSSA is e0/Km<<1."

We appreciate Reviewer's comment above. However, our calculation suggests that the relation $e_0/K_{\mathrm{M}} \ll 1$ in Reviewer's comment may not serve as a necessary condition of the sQSSA, due to the existence of the counterexample. The counterexample is the case that $e_0/K_{\mathrm{M}} \gtrsim 1$ and $e_0/s_0 \ll 1$, that is, $K_{\mathrm{M}} \lesssim e_0 \ll s_0$. It is straightforwardly satisfied that $e_0 \ll s_0 + K_{\mathrm{M}}$. For the sake of simplicity, further assume that the complex-to-product conversion rate equals zero and thus the system essentially reaches a steady state after some transient period of the complex formation. Because the "total" substrate concentration in this system remains s_0 over time, the exact solution of the complex concentration at the steady state is given by the tQSSA with s_0 and e_0. To this exact solution, applying the Padé approximant based on the above relation $e_0 \ll s_0 + K_{\mathrm{M}}$ now retrieves the sQSSA form of s_0 and e_0. Actually, in this case, the exact solution of the complex concentration is close to e_0, also consistent with the sQSSA result. Hence, we kindly note that the sQSSA may not necessarily imply the condition $e_0/K_{\mathrm{M}} \ll 1$ in Reviewer's comment.

Response to Reviewer #4

First of all, we are very thankful for Reviewer's valuable comments and suggestions. In accordance with these comments, we here offer a list of the changes made in the manuscript.

> *"Below I detail a series of Major and Minor concerns that need be addressed for this potentially important work to be accepted.*
>
> *Major Concerns*
>
> *Text S1 starts out detailing clearly the approach and reveals the first key assumption S7 to correctly derive results S8-S10. As the normalized discriminant >1, inequality S7 defines a plausibly wide range for the validity of the approximation. However, from then on there is much confusion.*
>
> *Firstly, the assumption S11 needs clarification and substantiation. i) Can the authors please demonstrate the time range over which this assumption is valid? This important as S12-13 give the impression that S11 is assumed to hold for all times. Can this assumption be demonstrated analytically when the sQSSA or tQSSA are valid?"*

We appreciate this careful question. In fact, in Text S5 *Preconditions of rate laws*, we showed that the assumption in Eq. (S11) is equivalent to the assumption $\varepsilon_2(\tau) \ll 1$ where $\varepsilon_2(\tau) \equiv \Delta_{\mathrm{tQ}}^{-3}(\tau)|[1+\bar{A}(\tau)-\bar{B}(\tau)]\bar{A}'(\tau)+[1+\bar{B}(\tau)-\bar{A}(\tau)]\bar{B}'(\tau)|$. For its derivation, refer to the description above Eq. (S31) in Text S5. Through the numerical simulation of Eqs. (S1) and (S40), we further showed that most physiologically-relevant conditions satisfy $\varepsilon_2(\tau) \leq 0.1$ **for the entire range of the simulation time**. These points have been reflected in the revised Texts S1 and S5, as follows (the revised part in bold):

> (Text S1) "In physiologically-relevant conditions, Eq. (S11) is **readily satisfied (see Text S5)**."
>
> (Text S5) "Next, we revisit another condition in Eq. (S11) ⋯ Regarding $\bar{C}(\tau)$ from Eqs. (S1) and (S40) in Text S7, ⋯ most of **our simulated,** physiologically-relevant conditions in Table S3 (88.1%) satisfy both

$\max_\tau[\varepsilon_1(\tau)] \le 0.1$ and $\max_\tau[\varepsilon_2(\tau)] \le 0.1$ **[i.e., $\boldsymbol{\varepsilon_1(\tau) \le 0.1}$ and $\boldsymbol{\varepsilon_2(\tau) \le 0.1}$ during the entire simulation time]** ⋯"

Beyond the above numerical simulations, we currently suppose that a constant term inside the square root of $\Delta_{\mathrm{tQ}}(\tau) = \sqrt{1 + 2[\bar{A}(\tau) + \bar{B}(\tau)] + [\bar{A}(\tau) - \bar{B}(\tau)]^2}$ [Eq. (3) in the main text], that is 1, may at least partially buffer the changes of $\bar{A}(\tau)$ and $\bar{B}(\tau)$ over time and thereby help $\Delta_{\mathrm{tQ}}(\tau)$ satisfy Eq. (S11), although establishing a more rigorous and sophisticated argument is clearly warranted in the future.

> *"ii) is S11 only assumed to hold over short durations in order to derive a local first order approximation? If so, this assumption should be made explicit and the derivation of S14 and S15 adapted accordingly. If not, please explain."*

We here answer Reviewer's above question. Briefly, Eq. (S11) is assumed to be valid at least over the short duration and this assumption gives rise to Eq. (S12) from Eq. (S10). On the other hand, Eq. (S15) is drawn from Eq. (S13) when Eq. (S32) is satisfied as explained in Text S5 *Preconditions of rate laws*, while Eq. (S14) is just the definition of a newly-introduced symbol in Eq. (S13). Thanks to Reviewer's valuable question, we have clarified our points with more detailed explanation in the revised Text S1, as follows (the revised part in bold):

> [Text S1, below Eq. (S11)] "**We then apply Eq. (S11) to Eq. (S10) and notice that $\boldsymbol{\int_{\tau'}^{\tau} \Delta_{\mathrm{tQ}}(\tau'')\mathrm{d}\tau'' \approx (\tau - \tau')\Delta_{\mathrm{tQ}}(\tau)}$ and $\boldsymbol{\int_{\tau_0}^{\tau} \Delta_{\mathrm{tQ}}(\tau')\,\mathrm{d}\tau' \approx (\tau - \tau_0)\Delta_{\mathrm{tQ}}(\tau_0)}$ for $\boldsymbol{\tau' \gtrsim \tau - \Delta_{\mathrm{tQ}}^{-1}(\tau)}$ and $\boldsymbol{\tau \lesssim \tau_0 + \Delta_{\mathrm{tQ}}^{-1}(\tau_0)}$, respectively. Subsequently, $\boldsymbol{\exp\left[-\int_{\tau'}^{\tau} \Delta_{\mathrm{tQ}}(\tau'')\mathrm{d}\tau''\right] = \exp\left[-\int_{\tau-\Delta_{\mathrm{tQ}}^{-1}(\tau)}^{\tau} \Delta_{\mathrm{tQ}}(\tau'')\mathrm{d}\tau'' - \int_{\tau'}^{\tau-\Delta_{\mathrm{tQ}}^{-1}(\tau)} \Delta_{\mathrm{tQ}}(\tau'')\mathrm{d}\tau''\right] \approx \exp\left[-1 - \int_{\tau'}^{\tau-\Delta_{\mathrm{tQ}}^{-1}(\tau)} \Delta_{\mathrm{tQ}}(\tau'')\mathrm{d}\tau''\right]}$, $\boldsymbol{\exp\left[-\int_{\tau_0}^{\tau} \Delta_{\mathrm{tQ}}(\tau')\,\mathrm{d}\tau'\right] = \exp\left[-\int_{\tau_0}^{\tau_0+\Delta_{\mathrm{tQ}}^{-1}(\tau_0)} \Delta_{\mathrm{tQ}}(\tau')\,\mathrm{d}\tau' - \int_{\tau_0+\Delta_{\mathrm{tQ}}^{-1}(\tau_0)}^{\tau} \Delta_{\mathrm{tQ}}(\tau')\,\mathrm{d}\tau'\right] \approx \exp\left[-1 - \int_{\tau_0+\Delta_{\mathrm{tQ}}^{-1}(\tau_0)}^{\tau} \Delta_{\mathrm{tQ}}(\tau')\,\mathrm{d}\tau'\right]}$, and the former and latter values become negligible for $\boldsymbol{\tau' \ll \tau - \Delta_{\mathrm{tQ}}^{-1}(\tau)}$ and $\boldsymbol{\tau \gg \tau_0 + \Delta_{\mathrm{tQ}}^{-1}(\tau_0)}$, respectively. Also, $\boldsymbol{\exp[-(\tau - \tau')\Delta_{\mathrm{tQ}}(\tau)]}$ becomes**

negligible for $\tau' \ll \tau - \Delta_{\mathrm{tQ}}^{-1}(\tau)$. Therefore, combined with Eq. (S11), Eq. (S10) for $\tau \gg \tau_0 + \Delta_{\mathrm{tQ}}^{-1}(\tau_0)$ is approximated as

$$\bar{C}(\tau) \approx \Delta_{\mathrm{tQ}}(\tau) \int_{-\infty}^{\tau} \bar{C}_{\mathrm{tQ}}(\tau') e^{-(\tau-\tau')\Delta_{\mathrm{tQ}}(\tau)} \mathrm{d}\tau', \qquad \text{(S12)}$$

where the right-hand side is not sensitive to the specific lower limit of τ' for the integral as long as this lower limit is $\ll \tau - \Delta_{\mathrm{tQ}}^{-1}(\tau)$."

[Text S1, below Eq. (S15)] "**The detailed condition for the validity of Eq. (S15) is provided in Text S5.**"

Next, the Reviewer asked the following questions:

"Assuming that you are planning to retain the current derivation, please explain i) why the second term in S10 does not contribute to S12, ii) and why the lower bound of the integral is -∞? ii) why the integral in S13 ranges from 0 to infinity?"

We appreciate Reviewer's valuable questions. As described in our revised Text S1 above, $\exp\left[-\int_{\tau_0}^{\tau} \Delta_{\mathrm{tQ}}(\tau')\,\mathrm{d}\tau'\right]$ exponentially decays out once $\tau \gg \tau_0 + \Delta_{\mathrm{tQ}}^{-1}(\tau_0)$, and therefore the second term in Eq. (S10) becomes relatively negligible in Eq. (S12) when $\tau \gg \tau_0 + \Delta_{\mathrm{tQ}}^{-1}(\tau_0)$. Next, $-\infty$ is taken for the lower bound of τ' in the integral in Eq. (S12) for the following reason: as described in the revised Text S1 above, $\exp\left[-\int_{\tau'}^{\tau} \Delta_{\mathrm{tQ}}(\tau'')\mathrm{d}\tau''\right]$ exponentially decays out once $\tau' \ll \tau - \Delta_{\mathrm{tQ}}^{-1}(\tau)$. Hence, the integral in Eq. (S12) is not sensitive to the specific lower bound of τ' as long as this lower bound is $\ll \tau - \Delta_{\mathrm{tQ}}^{-1}(\tau)$, and we therefore chose $-\infty$ for analytical convenience towards Eq. (S13). Lastly, the integral in Eq. (S13) runs from 0 to ∞ for the following reason: substituting $(\tau - \tau')\Delta_{\mathrm{tQ}}(\tau)$ with x straightforwardly gives the range of x in Eq. (S13) as $0 \leq x < \infty$, because $-\infty < \tau' \leq \tau$ in Eq. (S12).

These points, except the last one, have already been reflected in our revised Text S1 presented above. In addition, the last point has been included in the revised Text S1, as follows (the revised part in bold):

[Text S1, below Eq. (S12)] "The Taylor expansion $\bar{C}_{\mathrm{tQ}}(\tau') = \bar{C}_{\mathrm{tQ}}(\tau) - (\tau - \tau')\bar{C}'_{\mathrm{tQ}}(\tau) + (\tau - \tau')^2\bar{C}''_{\mathrm{tQ}}(\tau)/2 - \cdots$ **and the replacement of** $\boldsymbol{(\tau - \tau')\Delta_{\mathrm{tQ}}(\tau)}$ **by** $\boldsymbol{x}$ lead Eq. (S12) to ⋯"

Next, the Reviewer made the following comments:

"Given that S11 is assumed and can be demonstrated to be valid, can and should the derivation change and a simplified form of S10 be directly derived? The transition from S14 to S15 needs to be justified more explicitly."

We appreciate these comments, and our answers have already been given in other places above.

"Moreover, if S15 is key, can it not be derived from a reformulation of S8 in terms of c=C¯-C¯_tQ, e.g. dc/dt+(dC¯_tQ)/dt=-Δ_tQ·c"

This is a very insightful comment. We are indeed delighted with the possibility that Reviewer's reformulation of Eq. (S8) above may suggest a more straight, alternative route to Eq. (S15). Yet, in our current manuscript, we prefer the existing route via Eq. (S12) because Eq. (S12) reveals the interpretable trajectorial structure of the relaxation dynamics of complex formation with the rich potential for future extension. Nevertheless, we fully agree to Reviewer's suggestion on the possible presence of an alternative route to Eq. (S15).

"Following the derivation and validating the various assumptions made along the way is hard enough for a particular case. I would therefore ask that the authors first do so for the MM case and either move the stochastic case to a separate paper, or clearly and explicitly detail the derivation for this case, rather than just outline it. Just like the validity of the tQSSA was demonstrated gradually for different models, so should the new approximation."

We are thankful for Reviewer's constructive suggestion. As we elaborated above, the revised Texts S1–S5 would now provide sufficient details for the understanding of our formulation. In addition, we kindly note that the separation of the stochastic case to another paper might not be much feasible because the example systems for the biological applications of the ETS in our main text (Sections *Autogenous control* and

Rhythmic degradation of circadian proteins) require both deterministic and stochastic formulations, as evident in their descriptions.

> *"Minor Concern*
> *The figures should match the text. In particular, Figure 1A should detail the additional steps mentioned in the text (lines 120-123)"*

We suppose that the "additional steps" in Reviewer's comment specifically mean "$k_\delta \equiv k_\mathrm{d} + r_\mathrm{c} + k_\mathrm{loc} + k_\mathrm{dlt}$ where k_d, k_loc, and k_dlt stand for the dissociation, translocation, and dilution rates of AB, respectively, and r_c for the chemical conversion or translocation rate of A or B upon the formation of AB" in Lines 121–123. However, we are afraid that including all k_d, r_c, k_loc, and k_dlt in Fig. 1(a) can just make this figure complicated without clearer message delivery, because the definition of k_δ in Line 121 is simply for the conceptual generality that "k_δ is not limited to a dissociation event but encompasses all rate events to lower the level of AB" (Line 124). Hence, (*i*) depicting only k_d to emphasize a dissociation rate and (*ii*) lumping the other rates (i.e., r_c, k_loc, and k_dlt) to $k_\delta - k_\mathrm{d}$ followed by a question mark ("?") would suffice, as in the current Fig. 1(a). Of course, if the Reviewer suggests a better idea for Fig. 1(a), we will be happy to follow it.

Lastly, to the editorial question *"Have the authors made all data and (if applicable) computational code underlying the findings in their manuscript fully available"*, the Reviewer gave the answer that *"No: Data only appear to be provided in figure format. And I do not recall seeing a numerical methods section."*

First, we kindly note that the numerical method section is provided in Text S12 of the original manuscript, as declared as "Numerical simulation and data analysis methods are presented in Text S12" in Section *Materials and methods* in the main text. Besides, this *Materials and methods* section itself provides the summary of the key contents of Text S12. In addition, our computational codes are available at GitHub (https://github.com/rokt-lim/Generalized_Michaelis-Menten_rate_law), as noted in our previous submission package.

About the figures, the specific parameters for the model simulation and their literature sources are provided in Tables S1–S17, as stated in those figure captions of the original main text and Supporting Information. In the revised manuscript, we further provide

the specific values of the simulated individual data points in Figs. 2(d) and S7(e) through new Tables S9 and S18, respectively.
