# Supplementary material for: Generalized Michaelis–Menten rate law with time-varying molecular concentrations
Source: PLoS Comput Biol. 2023 Dec 11;19(12):e1011711. doi: 10.1371/journal.pcbi.1011711 (PMC10735182; doi:10.1371/journal.pcbi.1011711)
Supplement: Attachment — Submitted filename: PLoSCompMM-paper-review_v1 - (1).docx [file pcbi.1011711.s002.docx]

The manuscript titled "Generalized Michaelis-Menten rate law with time-varying molecular concentrations" presents a novel approach to model reduction in biochemical systems, called effective time-delay scheme (ETS), which generalizes the Quasi-Steady State Approximation (QSSA). The ETS introduces a time delay describing the effective relaxation time in complex formation into the QSSA equations and provides a more accurate description of the time courses of molecular processes than the simple QSSA in various examples.

Overall, the manuscript offers valuable insights into biochemical model reduction beyond the widely-used QSSA. However, some points need clarification to strengthen the results.

**Major**

1. Page 7, Line 184. The ETS returns to the QSSA in its form when the effective time delay is ignorable. Is the condition for ignoring the effective time delay consistent with the tQSSA validity condition?
2. Text S5. The applicability of the ETS and QSSA were compared with the area of the parameter regions where the validity conditions for the ETS (Eq. (S32)) and QSSA (Eq. (S34)) were satisfied, respectively. Does the validity region for the ETS cover the validity region for the QSSA? Please illustrate the validity regions to provide more information.

**Minor**

1. Figure 1. The model equations and parameters used to draw the graphs were not clearly explained.
2. Page 5, Line 198–209. The physical interpretation of the ETS is hard to understand. This part needs to be revised to improve readability.
3. Page 6, Line 151, Page 15, Line 460. Recent studies have shown that using the tQSSA (more specifically, Eq. (5)) is more accurate than the MM rate law in predicting drug clearance and drug-drug interaction mediated by enzyme induction (10.1111/cts.12804, 10.1002/cpt.2824).
4. Figure 2 (d). The explanation for Figure 2 (d) in the main text is insufficient. Moreover, Figure 2 (d) seems unnecessary to understand the analytical response time calculation. It can be moved to the Supporting Information.
5. Figure 4 (a), (b). Figure 4 (a) and (b) do not intuitively illustrate the comparison between the relative errors of parameter estimation based on QSSA and ETS. We suggest a heatmap or box plot for the relative error comparison.
6. In the ‘Rhythmic degradation of circadian proteins’ section, although the authors referred to Text S11 when they introduce the kinetic model (Line 368–369), it would be better to write the main model equations in the main text to understand the section.
7. Page 18, Line 531-534. The generalization of Eq. (7) to when the number of DNA binding sites is an arbitrary natural number, called the stochastic QSSA, has been derived in previous studies (10.1371/journal.pcbi.1005571, 10.1371/journal.pcbi.1008952). In addition, by comparing the stochastic QSSA with the deterministic QSSAs (i.e., tQSSA and sQSSA), studies have derived the validity conditions for using the deterministic QSSAs in stochastic models, which are generally stricter than those in deterministic models (10.1016/j.bpj.2014.06.012, 10.1371/journal.pcbi.1008952). Extending these studies using ETS would be an interesting candidate for the discussed future work.
8. Text S1 and S4. The main theoretical results of the paper are presented in these sections. These sections, or at least the summary of these sections, need to be provided in the main text.
9. Text S5. Eq. (S29) was derived from Eq. (S7) using Eq (S14), which was derived under the assumption of Eq. (S7). Is Eq. (S29) equivalent to Eq. (S7)?
10. Text S7. The calculation of the phase differences between the approximations (i.e., ETS, tQSSA, and sQSSA) and the actual time course of C is unclear.
11. Text S5. The derivation of “Delta_{tQ}^{-2} Delta’_{tQ} << 1” is unclear.
12. In TextS12, add subsection titles to improve readability.
